# When the earth shakes on fragile foundations: the Venezuelan earthquakes and two decades of public health system erosion

**DOI:** 10.1016/j.lana.2026.101666

**Published:** 2026-10-14

**Authors:** Alberto E. Paníz-Mondolfi, Carlos Chaccour, Fhabián S. Carrión-Nessi, Álvaro Acosta-Serrano, Omar Zambrano, Juan C. Gabaldón-Figueira, María-Eugenia Grillet

**Affiliations:** a Department of Pathology, Molecular and Cell-Based Medicine, Icahn School of Medicine at Mount Sinai, New York City, NY, USA; b Venezuelan Science Incubator, Barquisimeto, Venezuela; c Navarra Center for International Development, Institute for Culture and Society, Universidad de Navarra, Pamplona, Spain; d Department of Biological Sciences, University of Notre Dame, Notre Dame, IN, USA; e Eck Institute for Global Health, University of Notre Dame, Notre Dame, IN, USA; f Anova Policy Research, Caracas, Venezuela; g Department of Microbiology, Hospital de la Santa Creu i Sant Pau, Barcelona, Spain; h Instituto de Salud Global, Barcelona, Spain; i Centro de Investigación Biomédica en Red de Enfermedades Infecciosas, Instituto de Salud Carlos III, Madrid, Spain; j Laboratorio de Biología de Vectores y Parásitos, Instituto de Zoología y Ecología Tropical, Facultad de Ciencias, Universidad Central de Venezuela, Caracas, Venezuela

**Keywords:** Earthquake, Humanitarian crisis, Health, Venezuela

## Abstract

The two earthquakes that recently struck the northern coast of Venezuela will deepen a health crisis that has troubled the country for twenty years. They have exposed how weak its health infrastructure is, how scarce medicines are, how little epidemiological surveillance remains, and how long investment has been lacking. The humanitarian health crisis in Venezuela did not arise from armed conflict, as it has elsewhere. Its roots lie in decades of authoritarian rule and institutional decay, which have eroded public health capacity, research infrastructure, vaccination coverage and the control of infectious diseases in general. Because these weaknesses have accumulated, the earthquakes may well turn into an even graver humanitarian emergency. Recovery that lasts will not be secured by short-term international aid alone. It will require the rebuilding of accountable institutions able to govern transparently and to apply equitable health policies, so that the health system can regain its resilience.

## Introduction

On June 24, 2026, two exceptionally strong earthquakes (Mw 7.2 and Mw 7.5) struck the north–central coast of Venezuela just seconds apart, causing a significant devastation in seven states.^1^ This near-simultaneous sequence constitutes one of the most catastrophic seismic events in the country’s recent history. Two epicenters were identified, one in between Yaracuy and Carabobo states, and the second off the coast of La Guaira state, with the greatest destruction concentrated in the cities of Catia La Mar and Caraballeda, both in La Guaira. Within hours, the U.S. Geological Survey’s Global Earthquake Rapid Assessment for Response (PAGER) system estimated a 42% probability of between 10,000 and 100,000 fatalities, highlighting the anticipated severity of the disaster.^1^ At the time of the events, emergency services were overwhelmed by the scale of the demand, as well as disruptions in telecommunications, water, electricity, and transportation systems, which triggered an immediate international humanitarian response.^2^ As of August 10, 2026, the latest official reports document 6301 deaths, approximately 73,356 hospitalizations and 6462 rescued people.^3^ The number of victims could rise and get close to the lower end of the projection made by PAGER, given that debris removal efforts are still ongoing, having only reached 23.3% of the projected total.^3^ By August 12, 2026, according to the Pan American Health Organization (PAHO),^4^ 28 transitional camps had been established in La Guaira and Miranda states, and the Capital District, sheltering approximately 12,752 displaced people. Meanwhile, according to the Office for the Coordination of Humanitarian Affairs (OCHA),^3^ by August 14, 2026, more than 304,000 people affected by the earthquakes across the seven directly affected states had accessed essential services and humanitarian assistance (Fig. 1).

**Fig. 1:**
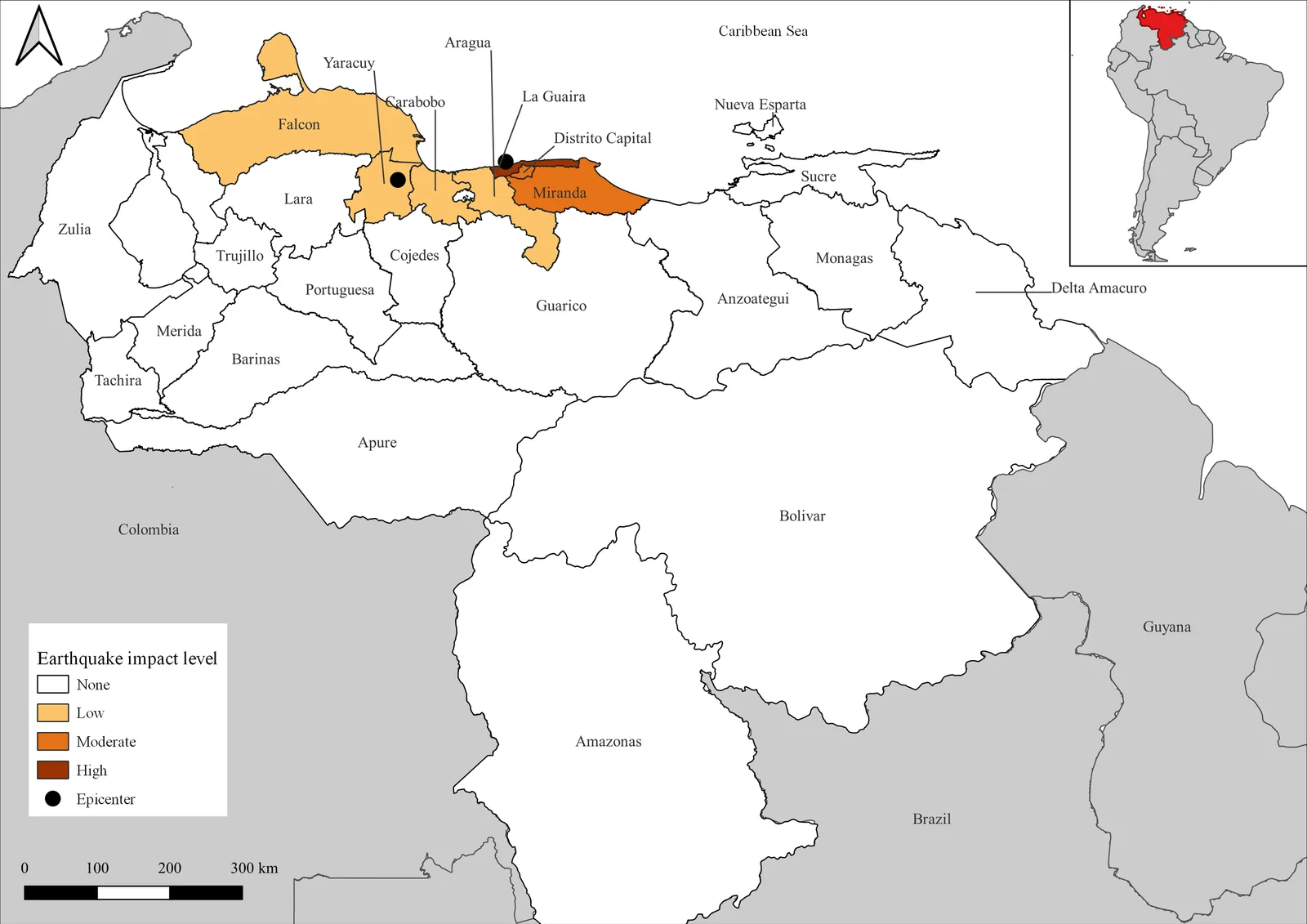
Map of Venezuela showing states affected by the two earthquakes, shaded by impact level. Inset (upper right) depicts Venezuela within South America. Map generated with QGIS software (https://www.qgis.org/es/site/).

The devastation and humanitarian consequences of the ongoing Venezuelan earthquakes cannot be explained solely by their geophysical magnitude. Rather, they are the culmination of more than two decades of institutional deterioration, during which health systems, public infrastructure, and scientific capacity eroded, leaving the country extremely vulnerable to major emergencies. While direct comparisons between events in different countries are complex, the remarkably lower toll of the similar Mw 7.4 earthquake that affected neighboring Colombia less than two months later, on August 10, 2026 (304 documented deaths, 4548 injuries, 426 missing persons, and 292,043 people affected)^5^^,^^6^ highlights Venezuela’s manifest institutional vulnerability.

Natural disasters rarely generate health crises on their own. They exacerbate pre-existing weaknesses, such as those in Venezuela: deficient health infrastructure, fragile supply chains, staff shortages, disruption of epidemiological surveillance, and a long-lasting lack of investment in science and public health. In Venezuela, these vulnerabilities had accumulated over decades, transforming a seismic event into the culminating crisis of a complex humanitarian emergency that was already underway.

## A health system with limited disaster preparedness

The June 2026 earthquakes severely impacted Venezuela’s health system, which already lacked capacity to respond to large-scale emergencies. Hospitals faced chronic shortages of personnel, supplies, equipment, and functional infrastructure. The 2024 National Hospital Survey, conducted by resident physicians across the country, found that only 40% of operating rooms in the 40 largest public hospitals selected for the evaluation were fully operational, while nearly three-quarters of emergency departments reported shortages of essential supplies.^7^ Similarly, HumVenezuela’s 2025 assessment revealed that La Guaira, the state most affected by the earthquake, was already experiencing critical deficiencies: medical personnel (44%), supplies (63%), medicines (78%), and equipment (77%).^8^ As early as September 2024, the National Hospital Survey already reported a 37% shortage of emergency supplies, widespread medicine scarcity, deteriorating service quality, staffing deficits, and limited diagnostic capacity.^7^ Many hospitals lacked contingency planning and could not sustain water, electricity, or medical gases for more than 72 h during outages. These vulnerabilities created a scenario of heightened exposure to disasters: when infrastructure fails, care collapses precisely as demand surges.

At the time of the earthquakes, the states, Capital District, La Guaira and Miranda had only 97 public health centers of different categories in operation (25 tertiary, 34 secondaries, 38 primary).^9^ However, PAHO’s rapid assessments of eight representative main public hospitals found structural damage in three and urgent needs for external support in all, with only 1220 beds available in total for an area with a combined population of approximately 6 million people.^9^ The reduced capacity accentuated the need for emergency supplies, trauma care, facility assessments, and operational coordination. The initial response required mobilization of national and international resources, including patient transfers to private hospitals and deployment of eleven international field hospitals.^2^ Emergency Medical Teams (EMTs) were coordinated on the field; but by August 12, 2026, only nine remained active across La Guaira, Caracas, and Miranda, while thirteen had demobilized.^4^ Seven weeks later, national and international operations continue to sustain emergency care while transitioning toward early recovery.

## Erosion of disaster preparedness and scientific infrastructure

The collapse of Venezuela’s health system has gone hand in hand with the deterioration of its scientific infrastructure,^10^^,^^11^ essential for disaster preparedness. By June 2026, the national seismic monitoring network had shrunk from nearly 300 stations in the 1990s to just a handful by 2026.^12^ Despite previous studies on seismic vulnerability,^13^ prevention and preparedness have been weakened, leaving the country without an effective early earthquake warning system: an institutional rather than technical failure. While early warning cannot prevent earthquakes, robust monitoring is vital for risk assessment, emergency coordination, and the rapid deployment of resources.^13^ This deterioration reflects a widespread decline in scientific investment,^11^ manifested in the weakening of epidemiological surveillance, laboratory networks, and university infrastructure.

## Infectious disease risk and the threat of resurgence

After a disaster, the greatest infectious disease threat is usually the resurgence of diseases that remain endemic, rather than the introduction of new pathogens, because the systems required to control them have been weakened. The recent seismic events occurred against the background of a prolonged and unresolved complex humanitarian emergency that had already damaged public health infrastructure in Venezuela and left the country more exposed to a resurgence of infectious diseases. Over the past decade several vaccine-preventable infectious diseases and vector-borne diseases have re-emerged there.^14^^,^^15^ Access to health care fell, surveillance systems weakened, prevention programs were interrupted, and the social determinants of health had all deteriorated; the re-emergences were the cumulative result of this situation.

As an example, Venezuela had major outbreaks of measles and diphtheria between 2017–2018,^14^ and the country’s immunization system has contracted dramatically over the past decade, with overall vaccination coverage declining since 2016. National investment on the immunization program fell from nearly US$50 million in 2014 to less than US$10 million in 2021.^16^ BCG coverage remained universal until 2018 but fell to 50% by 2025 (Fig. 2). IPV1 and YFV coverage declined to 30–35% in 2018, while DTP1 and DTP3 coverage decreased from ≥90% to 50–60% by 2025. Measles coverage peaked at 98% (MCV1, 2020) before halving, and MCV2 coverage remained below 60%. Pneumococcal and rotavirus control programs have been absent for over a decade. Overall, these trends reveal a progressive erosion of immunization coverage, with brief recoveries only around 2019–2020. By 2025, most vaccination coverage had fallen below the regional threshold of 80%, increasing the risk of a resurgence of preventable diseases. With support from PAHO and the humanitarian aid health cluster, it has been reported that between June 28 and August 3, 2026, 31,384 vaccine doses were administered in the affected states, including 28,100 doses in La Guaira.^4^ By August 12,^4^ 2026, three displaced families had arrived in Sucre, Bolívar, and Delta Amacuro, highlighting the urgent need to expand vaccination, strengthen surveillance, and prioritize prevention nationwide, not only in Zone 0. In the post-earthquake context, restoring coverage must extend beyond affected areas, with enhanced surveillance for early case detection and targeted vaccination in high-risk regions and age groups.

**Fig. 2:**
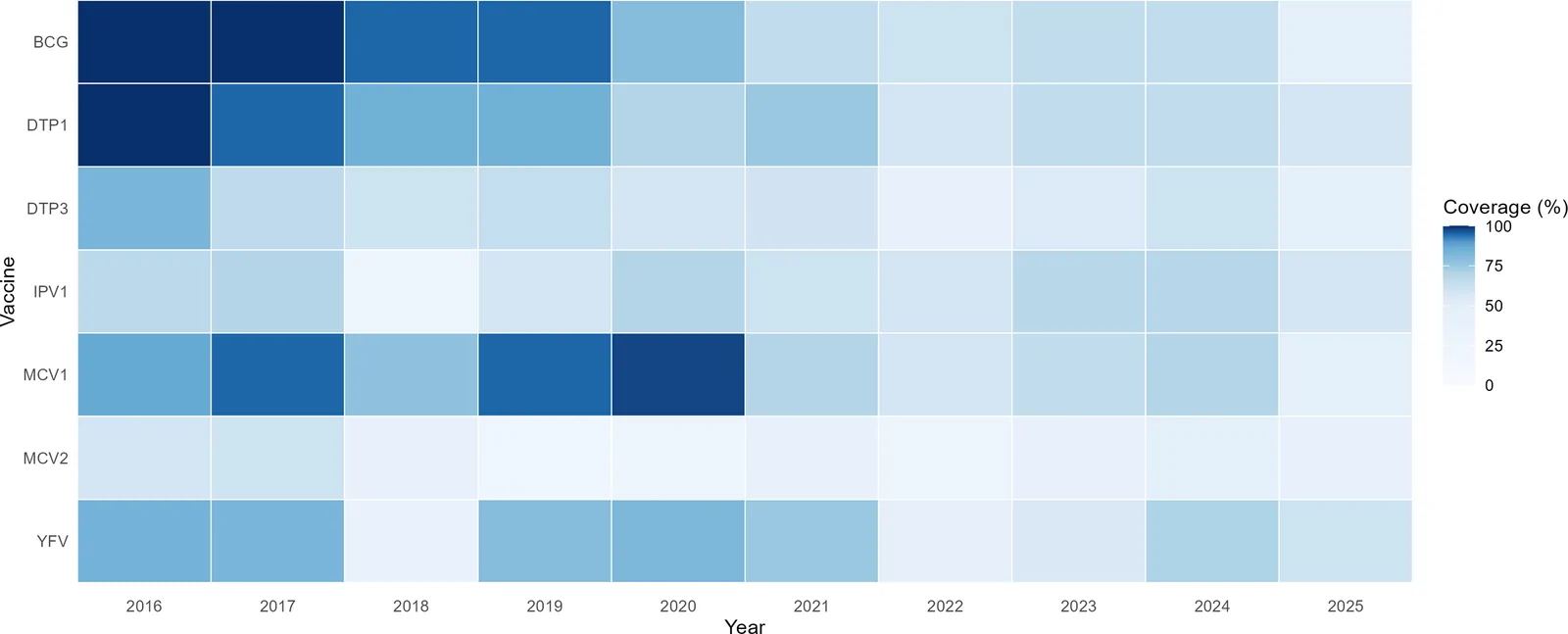
Heatmap showing vaccine coverage (%) in Venezuela from 2016 to 2025 for seven essential vaccines: BCG (tuberculosis), DTP1 and DTP3 (diphtheria, tetanus, and pertussis, first and third doses, respectively), IPV1 (polio, first dose), MCV1 and MCV2 (measles, first and second doses, respectively), and YFV (yellow fever). Coverage levels are color-coded to highlight declines and partial recoveries across the decade. Source: WHO/UNICEF estimates of national immunization coverage (https://worldhealthorg.shinyapps.io/wuenic-trends/).

Vector-borne diseases remain a particular concern, as several pose heightened risks to public health. In areas affected by the earthquakes, dengue virus, transmitted by *Aedes aegypti* mosquitoes, typically increases between September and December, and even more so with the potential strong El Niño event that is predicted to further amplify transmission toward the end of 2026 in the northern coastal states of the country.^17^ Yellow fever, which has already re-emerged since 2025 with cases registered in Aragua, a state relatively close to the affected area,^18^ matching a low national vaccination coverage.

Following an earthquake, multiple other infectious diseases also tend to emerge, including respiratory, gastrointestinal, and skin/soft tissue infections pathogens.^19^ Moreover, exposure to dust and debris, overcrowding in shelters, the collapse of health services, and the scarcity of potable water and sanitation facilities further drive the transmission of tetanus, diphtheria, measles, and other respiratory infections, including pneumococcal disease. As an example, the concentration of large numbers of people in temporary and overcrowded shelters heightens opportunities for the transmission of tuberculosis.^20^ At the same time, disruption of waste management, food storage, and sanitation favors rodent population growth, increasing the risk of rodent-borne pathogens like *Leptospira* spp.^21^

Although human remains are generally considered to present a relatively low risk for transmission of epidemic pathogens, their prompt recovery, identification and appropriate containment remain essential.^22^ Delays in these efforts, as witnessed to date, can lead to advanced decomposition, potentially compromising forensic identification and making the recovery and documentation of victims considerably more difficult. In the aftermath of the earthquakes in Venezuela, however, these efforts have been severely constrained by the disruption of essential infrastructure, overcrowded morgue facilities, and critical shortages of resources such as body bags required for management of human remains.^23^

Beyond infectious diseases, the humanitarian crisis has affected maternal and child health. Approximately 11% of all children under five year-old in Venezuela are estimated to be moderately or severely malnourished,^24^ and as of 2022, infant mortality has been estimated between 20 and 25 deaths per 1000 livebirths, an increase of 30–60% compared with 2011 figures.^25^ Similarly, maternal mortality increased in 173% between 2000 and 2023, reaching 227 deaths per 100,000 livebirths, roughly three times the regional figure.^26^ Severe food insecurity, malnutrition and the disruption of essential health care services have worsened health outcomes among vulnerable populations, including children and pregnant women.^27^ Reliable national indicators remain limited due to a lack of transparency and persistent uncertainty in health information, but the evidence available points to persistent problems in nutrition, maternal care and access to preventive services.

Indeed, in Venezuela, the chronic absence of epidemiological bulletins for a decade (2016–2026)^15^ was, until very recently, a major obstacle to an effective response. Therefore, surveillance systems in the country must continue to be strengthened to promptly detect outbreaks, validate early warnings, and guide emergency recovery.

## Preserving essential health services and the scale of recovery beyond immediate damage

Trauma care was the immediate priority after the earthquakes, but the disaster also threatened the continuity of care for people who depend on long-term treatment for chronic infectious diseases. The Global Fund recognized this risk and approved an emergency allocation of US$1.77 million less than one month after the disaster, so that HIV and tuberculosis services in Venezuela could continue without interruption.^28^

The emergency response is implemented through the United Nations Development Programme. Its aim was to maintain treatment while diagnostic capacity, laboratory services, community outreach, prevention programs and telemedicine support were restored in the affected areas. The Global Fund estimated that about 31,000 people living with HIV and 2400 people receiving tuberculosis treatment were at risk of interruptions in their care if no rapid intervention was made.^28^

This response draws attention to a dimension of disaster medicine that is frequently overlooked: continuity of care for chronic infectious diseases matters as much as the management of acute trauma. If antiretroviral therapy, tuberculosis treatment or diagnostic services are interrupted, the consequences for the individual patient are immediate, and the risks of transmission, treatment failure and drug resistance also rise.

The rapid mobilization of international aid underlines the importance of global health partnerships during emergencies. Following the earthquakes, the demand for medical care surged among millions of people already in need, overwhelming a system with structural deficiencies and limited financial access, where out-of-pocket payments often supplemented public services.^29^ On July 10, 2026, the United Nations announced an additional allocation of US$298 million to the Humanitarian Response Plan already in place in the country, intended to address the multisectoral needs of 1.3 million people over six months, including psychosocial support and treatment for communicable and non-communicable diseases in temporary camps.^2^

But the consequences of the earthquakes extend far beyond immediate destruction. Long-term recovery in Venezuela requires distinguishing between direct physical damage and the need for structural and institutional reconstruction. Direct damage reflects the value of destroyed property, while reconstruction costs encompass the broader resources and political will needed to restore infrastructure, services, and institutional capacity. An analytical model developed by the United Nations Office for Disaster Risk Reduction, in collaboration with Ingeniar CAD/CAE, estimated direct damage to buildings at approximately US$24 billion and infrastructure losses at an additional US$13 billion, for an event with a return period of approximately 180 years.^30^

These projections should not ignore a critical reality: Venezuela’s health system cannot be rebuilt by repairing hospitals alone. Emergency financing may temporarily offset vulnerabilities, but resilience depends on sustained investment in health care delivery, laboratory infrastructure, supply chains, surveillance systems, and the workforce required to maintain essential services before another disaster strikes. Long-term recovery must also restore core public health functions, disease surveillance, supply chains, emergency preparedness, and scientific institutions capable of supporting evidence-based decision making.

## Conclusion

The earthquakes of June 2026 in Venezuela exposed the fragility of a health system eroded by institutional decay, chronic underinvestment, and a loss of capacity. These events did not create new vulnerabilities but rather revealed and exacerbated existing ones. Over the subsequent 6–24 months, PAHO, the UNDP, national institutions, and international partners must leverage the recovery period to rebuild essential systems. Priority must be given to rehabilitating damaged facilities and ensuring continuity of care while restoring hospital services, basic utilities, supply chains, laboratories, and epidemiologic surveillance systems. Progress should be assessed at the 6-, 12-, and 24-month marks using indicators such as hospital functionality, continuity of treatment and vaccination, diagnostic reliability, and the operational status of emergency plans, including seismic monitoring.

Long-term recovery requires rebuilding the foundations of public health and strengthening governance to ensure transparency and equity. This involves having functional facilities, effective supply chains, robust surveillance systems, laboratory capacity, sufficient health personnel, and institutions capable of delivering effective care. While natural phenomena cannot be prevented, their impact can be mitigated. Critically, Venezuela’s experience demonstrates that pre-existing institutional weaknesses are often as damaging as the natural disaster that leaves them in evidence.

## Contributors

AEPM and MEG conceptualized this viewpoint, searched for data and published works, and drafted the original manuscript. CC, FSCN, AAS, OZ and JCGF, reviewed and edited the manuscript. All authors read, revised, and approved the manuscript.

## Editor note

The Lancet Group takes a neutral position with respect to territorial claims in published maps and institutional affiliations.

## Declaration of interests

The authors declare that they have no conflicts of interest.
